# The value of dynamic elastomeric fabric orthoses in the management of a complex hypermobile Ehlers‐Danlos syndrome patient: A case report

**DOI:** 10.1002/ccr3.6821

**Published:** 2023-01-16

**Authors:** Anna Higo, Gemma Pearce, Shea Palmer, Louise Grant

**Affiliations:** ^1^ Centre for Healthcare Research Coventry University Coventry UK; ^2^ Physiocure Physiotherapy Clinic Leeds UK; ^3^ Centre for Care Excellence Coventry University and University Hospitals Coventry & Warwickshire NHS Trust Coventry UK

**Keywords:** dynamic elastomeric fabric orthoses, hip dysplasia, hypermobile Ehlers‐Danlos syndrome, hypermobility, joint subluxation, periacetabular osteotomy, physiotherapy, Postural Tachycardia syndrome

## Abstract

The use of dynamic elastomeric fabric orthoses is examined in a young woman with hypermobile Ehlers‐Danlos syndrome (hEDS) referred for physiotherapy with hip dysplasia, prior to a right periacetabular osteotomy. Dynamic elastomeric fabric orthoses plus rigorous subjective examination, therapists' listening skills, and patient‐centered goals were useful for this hEDS patient.

## 1 INTRODUCTION

The Ehlers‐Danlos syndromes (EDS) are a group of heritable connective tissue disorders, which have 13 subtypes as defined by the 2017 International Classification.^1^ EDS is characterized by joint hypermobility, connective tissue fragility, and skin extensibility, with widespread manifestations in the skin, ligaments, joints, blood vessels, and internal organs; and the clinical spectrum varies from mild skin and joint hyperlaxity to severe and life‐threatening vascular complications.^1^ Hypermobile EDS (hEDS) is the most common subtype and is associated with pain, activity limitations, and participation restrictions.^2^ hEDS, currently, has no genetic marker and is diagnosed through a clinical examination using the 2017 International Classification.^1^, ^3^ Additional consideration must also be given to the comorbidities that exist alongside the main condition due to its multisystemic nature. These include Postural Tachycardia Syndrome (PoTS),^4^ Mast Cell Activation Syndrome (MCAS),^5^ gastrointestinal issues, and bladder and bowel problems.^6^ These comorbidities can play an important role when it comes to rehabilitation planning.^7^ Comorbidities are associated with worse health outcomes, more complex clinical management, and increased healthcare costs.^8^

This group of patients is poorly recognized and poorly understood, as highlighted in recent studies of healthcare experiences.^9^, ^10^, ^11^ Physiotherapists play a central role in management, yet many practitioners fail to recognize the complexity of these patients and that inappropriate treatment may be detrimental to their management.^7^, ^12^ Existing studies have highlighted the psychological impact of this condition and recommend early detection to assist optimum management.^9^, ^13^ Healthcare practitioners therefore need increased awareness of hEDS.

Dynamic Elastomeric Fabric Orthoses (DEFO) are fabric elastomeric orthotic garments that have strategic reinforcement biomechanical paneling.^14^, ^15^ Therefore, they may help people with hEDS by exerting a customized paratonic torsional, compressive and supportive effect that could positively influence alignment biomechanics, and neuromuscular activity specific to the individual and their body segments.^14^ DEFOs are different from traditional braces, which may restrict movement and prevent muscle activity.^15^ They can also be individually tailored to the patient's needs with specific alterations. Case reports have shown their successful use in improving form and/or function in a variety of patients with movement control problems.^15^, ^16^ A study of six hEDS patients and six matched controls demonstrated that postural control impairment was partially offset by wearing somatosensory orthoses.^17^ Simmonds et al.^18^ suggested pain, fatigue, and fear of injury are common barriers to exercise. Therefore, in the hEDS patient who commonly suffers with pain, impaired proprioception, poor muscle control, increased tissue elasticity,^19^, ^20^ and reduced function; DEFO could be considered as the first line of treatment.

Postural Tachycardia Syndrome, a comorbidity found in EDS,^21^ is defined as a clinical syndrome lasting at least 6 months that is characterized by: (1) an increase in heart rate ≥30 beats per minute within 5–10 min of quiet standing or upright tilt (or ≥40 beats per minute in individuals 12–19 years of age); (2) the absence of orthostatic hypotension (>20 mm Hg drop in systolic blood pressure); and (3) frequent symptoms that occur with standing such as lightheadedness, palpitations, tremulousness, generalized weakness, blurred vision, exercise intolerance, and fatigue.^22^ Compression garments have been highlighted as a tool in the management of PoTS,^23^, ^24^ therefore the compressive effects of DEFOs might help to reduce symptoms. The management of hEDS must focus on treatment interventions that allow these patients to function, addressing “injury prevention and symptom amelioration rather than a cure”.^25^

## 2 CASE HISTORY AND EXAMINATION

The patient was a 22‐year‐old female Caucasian (height 160 cm, weight 70 kg) living in the United Kingdom with bilateral developmental dysplasia of the hip (DDH) with a radiographic Lateral Centre Edge Angle (LCEA) of 15° on the right and 17°. An LCEA of <20° is considered to be dysplastic.^26^ She had been referred for preoperative physiotherapy by her hip surgeon, in preparation for a right hip periacetabular osteotomy.^27^ The right hip operation was scheduled in 16 weeks' time and the left hip operation a year later; providing surgical outcomes were satisfactory on the right side. The patient has formally consented to her case being published, having considered and approved this manuscript.

### 2.1 Subjective history

The patient completed self‐reported questionnaires, namely the international Hip Outcome Tool (iHOT‐33),^28^ EuroQol 5 dimensions (EQ‐5D‐5L),^29^ and numerical pain rating scale (NPRS)^30^ as displayed in Table 1. She described right hip pain with a 9‐month history, in a “C‐sign distribution” (groin, lateral hip, and buttock).^31^ Pain was reported at night and affected all activities, including sitting and standing (both limited to 20 min), which affected her being able to work in her office job. Yoga used to be her favorite form of exercise as she could adopt to the positions easily but that was also now painful. She tried physiotherapy locally for the last 8 months, but the exercises caused her hip pain to flare up, so she stopped doing any exercise. Physiotherapy exercises previously prescribed had aimed to improve gluteal strength.^32^

**TABLE 1:** Self‐reported outcome measure data

Self‐reported outcome measures	Initial assessment	8‐week follow‐up	16‐week follow‐up
iHOT‐33	16	31	47
EQ‐5D‐5L			
Mobility	4	3	3
Self‐care	4	3	3
Usual activities	4	3	3
Pain/discomfort	4	3	2
Anxiety/depression	3	3	2
EQ‐5D‐5L scale of health	40	47	52
NPRS	8	7	6.5

*Note*: iHOT‐33—international Hip Outcome Tool‐33 (0 = severe problems, 100 = no problems); EQ‐5D‐5L—5 dimensional, 5 level Quality of Life instrument (1 = no problems, 5 = extreme problems); EQ‐5D‐5L scale of health (0 = worst health you can imagine, 100 = best health you can imagine); NPRS – Numerical Pain Rating Scale (0 = no pain, 10 = pain as bad as you can imagine).

In addition to her DDH, she reported endometriosis, polycystic ovaries, hay fever, irritable bowel syndrome, heartburn, reflux, depression, and chronic fatigue. Medications taken were Codeine for pain relief 15 mg once a day, Paracetamol 200 mg, 2–3 times a day, Cymbalta 60 mg once a day, and Medroxyprogesterone 25 mg once a day.

### 2.2 Objective examination

The patient walked into the clinic independently mobile with no walking aid but had a slight bilateral Trendelenberg walking pattern and stood predominantly weight‐bearing through the left leg. Initial observations were that the patient stood in a sway back posture (viewed from a lateral aspect, the greater trochanter of her hips bilaterally was positioned anterior to both the lateral aspect of the shoulder and lateral malleolus), with hyperextended knees and bilaterally pronated feet. On sitting she had her legs tightly crossed, with her feet tucked around one of the chair legs. The Beighton's hypermobility scale was 7/9^3^, ^33^, ^34^ with negative findings only for her thumb joints. The scale was explained to the patient and she reported this had never been performed on her before and she had never been told about hEDS in any of her past healthcare experiences. A preliminary hypermobility discussion ensued and it was explained that some patients experience a range of common comorbidities. This resulted in the patient disclosing that she bruised easily, had unexplained stretch marks on the skin around her trunk, widened atrophic scars from wounds from childhood injuries (from falling over), suffered recurrent ankle sprains, previous left wrist subluxations, allergies, and that she experienced dizziness and lightheadedness upon standing (and on a few occasions had fainted and fallen). She expressed how she often did not tell people about all of her symptoms as she was afraid of not being believed and being labeled as a hypochondriac.

Isometric muscle strength tests of the hips using a Hand‐Held Dynamometer (HHD) were carried out to record preoperative benchmark measurements. The testing protocol used was as per the Hip Arthroscopy Pre‐habilitation Intervention study^35^ with the addition of hip internal rotation in prone described by Thorborg et al.^36^ Measurements are presented in Table 2. Hip flexion and hip extension strength tests were not recorded due to the patient's high pain level with these movements. Functional movements such as squatting, bridges, one‐leg balance, single leg squat, step‐ups, and gait were observed, alongside the assessment of proprioception and motor control. All of these highlighted significant weakness, poor proprioception, and poor neuromuscular control. Due to her history of recurrent ankle sprains, clinical observation of bilateral foot pronation and the understanding of the importance of ankle push‐off function in reducing pressures through the anterior hip joint,^37^ the feet and ankles were assessed in detail. Passive tests of the ankles and feet showed the excessive range of motion and ligament laxity.^38^ Joint hypermobility can be found in joints outside of the Beighton score so a whole‐body approach was adopted.^20^, ^34^ Single‐leg calf raise ability, in supported one‐leg stance, was a total of five repetitions on each side before fatigue set in, which is a significantly lower value than would be expected in an average healthy adult.^39^ It is normal following periacetabular osteotomy surgery for a patient to use crutches and to have a weight restriction through the operated leg^40^; thus, the nonoperated leg must be of sufficient strength to cope with this.

**TABLE 2:** Hand‐held dynamometry values measured in pounds (lbs)

	Initial assessment	8‐week follow‐up	16‐week follow‐up
Hip abduction‐ left	11.8	15.6	22.3
Hip abduction‐right	10.3	13.1	19.7
Hip adduction‐left	8.7	14.0	18.3
Hip adduction‐right	9.1	15.6	19.2
Hip internal rotation‐left	7.7	10.6	12.9
Hip internal rotation‐right	8.4	11.5	13.4
Hip external rotation‐left	10.9	16.8	21.5
Hip external rotation‐right	9.8	14.7	17.0

### 2.3 Problem list

- Poor proprioceptive awareness
- Unable to improve preoperative function and strength, due to pain
- Safety concerns with dizziness, lightheadedness, and fainting episodes, combined with reduced mobility
- Low mood due to decreased function

### 2.4 Differential diagnosis, investigations, and treatment

After discussion, and with the patient's explicit consent, a letter was written to their Primary Healthcare Physician, General Practitioner (GP), to request a referral to a specialist Rheumatologist who dealt regularly with EDS and other connective tissue disorders. Following a thorough Rheumatology assessment and screening to exclude any other possible diagnoses, the specialist Rheumatologist diagnosed the patient with hEDS using the 2017 International Classification^1^, ^3^ and they also suspected PoTS. Therefore, they made an onward referral to a Cardiologist and for a tilt table test.^4^, ^22^, ^23^ The consultant Rheumatologist also liaised with the patient's orthopedic hip surgeon as there is a strong correlation between joint hypermobility and hip dysplasia.^44^ The patient reported that she felt listened to, felt the value of understanding her own body better, and felt having a “label” helped her explain her problems to family, friends, and work colleagues.^9^ She shared with us that she had previously “lost her faith” in physiotherapy, as exercises prescribed by past practitioners had caused pain exacerbation, so it was important for us to build trust and a relationship to help find a way of improving her strength, proprioception, and function.

To address the issues listed on the problem list we suggested a trial of DEFO leggings, which not only provide compression to aid blood pressure in the legs^23^ but also provide proprioceptive feedback to aid her postural control. With this patient, we wanted to reduce her sway back posture and knee hyperextension as we found that this decreased her pain. Sway back posture has been reported to increase the joint pressure around the anterior acetabulum, the area that is often inadequately covered by the hip socket in DDH.^37^ The patient's decreased awareness of her body posture meant she was unable to maintain the position, which decreased her pain; therefore, we wanted to assess to see whether a DEFO, in the form of leggings, which also encompassed the hip and pelvic region, could assist in reducing her sway back posture. Core control, hip, lower limb, breathing, and proprioceptive exercises^41^ were tailored to her needs so that they were manageable; paced to accommodate her pain, chronic fatigue, and dizziness; and were relevant to helping her function postoperatively. In a systematic review of exercise programs for hEDS patients, a period of 4–8 weeks was commonly reported in studies.^41^ Our exercise program had a longer timespan (16 weeks) as it was dictated by the planned surgery date.

### 2.5 Outcome and follow‐up

**FIGURE 1:**
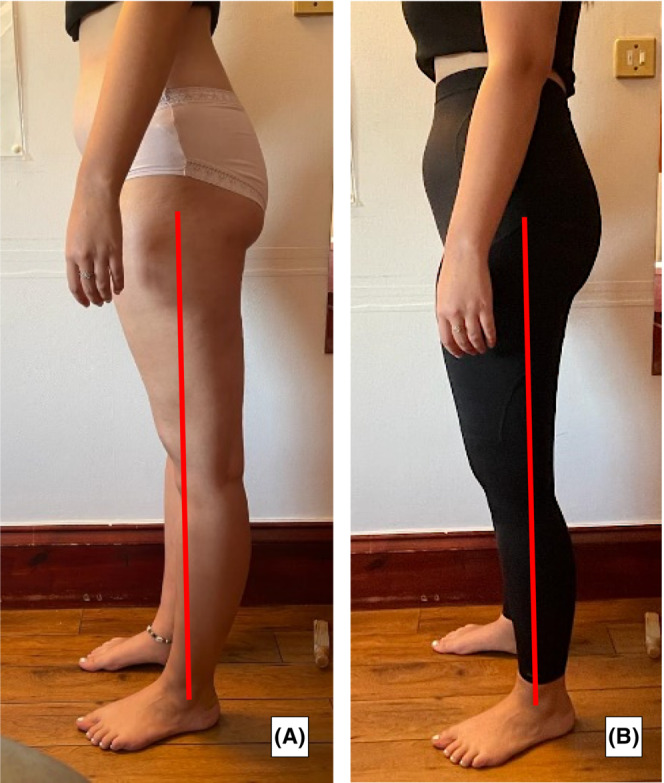
Lateral view photograph of case study patient posture. No dynamic fabric orthosis (A) and with dynamic fabric orthosis (B). Red line is drawn from midpoint of the lateral tip of greater trochanter to the midpoint of the lateral malleolus

Patient measurements were reassessed at 8 and 16 weeks, postintervention (Tables 1 and 2). Both subjective and objective outcome measurements showed positive improvements with the iHOT‐33 score increasing by 31 points. The minimal clinically important difference (MCID) of the iHOT‐33 is 6 points.^28^ The patient felt less pain and reported the DEFO made her feel more coordinated and stable. This resulted in her being able to engage in her physiotherapy exercises to prepare for hip surgery. Increases in muscle strength were demonstrated by HHD tests with her hip abductors, hip adductors, hip internal, and hip external rotators. Reduction in her symptoms of light‐headedness, dizziness, and palpitations were notable and promising in respect of improvements to her safety and prevention of falls. Figure 1 are photographs showing the patient without the orthoses and their sway back posture and a comparative photograph to show her posture changes while wearing the orthoses. Her posture in standing while wearing the orthotics was significantly more vertically aligned. These improvements also carried over into her walking posture; and most importantly, resulted in decreased pain and heightened body awareness.

## 3 DISCUSSION

This report highlights a novel approach to finding a practical solution to facilitate preoperative physiotherapy prior to hip surgery for DDH in a patient with multiple problems that were presenting as a barrier to exercise engagement. The use of DEFO garments could be considered as an intervention to allow improved function in people with hEDS and PoTs. A garment that encompasses the abdomen and lower body, and exerts a compressive force, has previously been shown to reduce heart rate and improve PoTS symptoms in adult patients.^42^ Reduced lower limb proprioception is a common problem in hEDS patients^43^ and joint hypermobility has been noted to be a key factor in hip dysplasia^44^; thus, an understanding of these elements can help support this group of patients. No previously published case studies have investigated the role of DEFO in this condition, therefore no direct comparisons can be made. This case study builds on Dupuy et al.'s pilot study improving postural control with DEFO in people with hEDS.^17^ Fatigue is a common debilitating complaint associated with PoTs,^4^, ^45^ and emerging evidence supports the use of compression garments to improve fatigue.^46^

We have highlighted the multisystemic nature of hEDS, and the importance of a thorough subjective examination, particularly the past medical history and the need for appropriate multisystemic questioning.^38^ Our case report also emphasizes the importance of recognizing when the timely onward referral is required to suitable medical specialists. hEDS patients have often had a long journey to diagnosis, with published research reporting a mean of 14 years elapsing between the first clinical manifestations and the actual diagnosis.^47^ Patients therefore often experience depression and anxiety after years of not being listened to and being discredited.^20^ To finally have someone that listens and subsequently acknowledges their multiple issues can often be the first step in their recovery.

In the example of the patient used in this case study, the inappropriate treatment led to pain‐related fear, fear‐avoidance, and further deconditioning. This is unfortunately common in hEDS patients.^48^ Pain‐related fear occurs when situations that are related to pain are seen as a main threat.^49^ This can be a fear of movement, fear of injury or reinjury, or fear of pain.^50^ Avoidance behavior is defined as postponing or preventing a potentially aversive situation from occurring.^51^ Such behavior will lead to a diminished quality of life as the patient has difficulties in physical functioning, mood, and social functioning.^52^ hEDS management overlaps with that of Hypermobile Spectrum Disorder (HSD),^11^ so these findings can be useful for HSD patients too. The majority of patients with HSD/hEDS have a heightened fear of movement^53^ and increased vulnerability to injury.^38^ Long‐term physical inactivity leads to physical deconditioning,^54^ including decreased muscle strength and cardio‐respiratory fitness. Despite studies identifying kinesiophobia as a possible barrier to exercise,^53^ the mainstay of treatment for HSD/hEDs is exercise and pain management.^41^ Literature conveys that these patients already have higher levels of anxiety,^55^ which may also have a dysautonomia element with symptoms such as hyperventilation, nausea, and light‐headedness, which are significantly more common in HSD/hEDS patients.^56^ Van Meulenbroek et al.^57^ found that adolescents with asymptomatic generalized joint hypermobility had the same level of physical functioning compared with non‐hypermobile controls. The lower levels of physical functioning observed in adolescents with HSD/hEDS could therefore not only be explained by the presence of generalized joint hypermobility; other issues need to be considered. Within the clinical practice, treatment needs to address other factors such as pain, fatigue, multisystemic dysfunction, loss of postural control, and pain‐related fear.^47^, ^50^

Via the use of the DEFO, we were able to tackle the key factors on this patient's problem list and bring about positive changes, enabling them with strategies to overcome barriers to exercise. It is important to note that there were no adverse effects reported by the patient on wearing the DEFO. The orthoses used in this case study were purchased from DM Orthotics Ltd, which supply Asia, North America, New Zealand, Australia, Europe, and the United Kingdom. There are other manufacturers of lycra orthoses such as Second Skin and Jobskin Ltd. We are unable to comment on these products from other manufacturers as they may differ from the DM Orthotic orthoses we used. Considering appropriate DEFOs for hEDS/HSD patients, for whom diagnostic challenges may arise, could provide the individual with a beneficial level of support. This in turn could decrease pain, improve function and increase confidence in their ability. This confidence might allow them to begin a carefully graduated, patient‐specific strengthening program, thus minimizing fear‐avoidance and potential injury; and in this specific case report, prepare physically for upcoming major surgery. This case study supports previous case‐control study evidence that DEFO might improve postural control in people with hEDS.^17^ Not only can the garments be used for the musculoskeletal system, but as this case indicates, they are also potentially beneficial in addressing the multisystemic nature of HSD/hEDS by reducing PoTS symptoms that in this instance were a safety concern with imminent orthopedic surgery.^58^ It was paramount in this case to ensure the patient had a reduced risk of fainting and falling to avoid undue stress to her periacetabular osteotomy and the newly forming bony union.^40^ This case study demonstrates the need for further robust studies into the use of these orthoses for individual joint problems, multiple joint issues, chronic fatigue, and dysautonomia.

## AUTHOR CONTRIBUTIONS

**Anna Higo:** Conceptualization; data curation; formal analysis; investigation; methodology; project administration; resources; software; writing – original draft; writing – review and editing. **Gemma Pearce:** Supervision; validation; writing – review and editing. **Shea Palmer:** Supervision; validation; writing – review and editing. **Louise Grant:** Visualization; writing – original draft; writing – review and editing.

## CONFLICT OF INTEREST

The authors have no conflicts of interest to disclose. The patient paid for her physiotherapy treatment and orthoses. This is an independent case study.

## CONSENT

Written informed consent was obtained from the patient to publish this report in accordance with the journal's patient consent policy.

## Data Availability

The data used to support the findings of this study are included in the article.
